# Socio-demographic characteristics and dietary pattern of community-dwelling adults in Abia State, Nigeria

**DOI:** 10.4314/gmj.v57i3.12

**Published:** 2023-09

**Authors:** Patricia O Ukegbu, Beulah Ortutu, Uche P Chinaza, Alice Ojwang

**Affiliations:** 1 Department of Human Nutrition and Dietetics, Michael Okpara University of Agriculture, Umudike, Abia State, PMB 7267, Umuahia, Nigeria; 2 Department of Human Nutrition and Dietetics, The Technical University of Kenya, Nairobi, Kenya

**Keywords:** Dietary patterns, PCA, Food Frequency, adults, Nigeria

## Abstract

**Objective:**

Identification of dietary patterns and their association with socio-demographic factors.

**Design:**

Community-based cross-sectional study design

**Setting:**

Urban and rural communities in Abia State, Nigeria

**Participants:**

Eight hundred and sixty-eight (868) male and female adults aged 20 to 59 years

**Methods:**

Identification of Dietary patterns (DP) by Principal Component Analysis (PCA) based on the consumption of 10 food groups, assessed using a 7-day qualitative food frequency questionnaire. Bivariate and multivariate logistic regression analyses evaluated the association between identified patterns and socio-economic factors.

**Results:**

Two dietary patterns ‘traditional and convenience DPs were identified, explaining 52% of the total variance. The traditional DP was loaded with starchy staples, vegetable soups/sauces, and animal proteins. The convenience DP was characterised by high factor loading of processed cereals, carbonated drinks and alcoholic beverages. Larger households (>3) had lower odds of adhering to high traditional DP [AOR =0.633; 95% CI (0.429-0.934); p = 0.021]. Females [AOR =1.586; 95% CI (1.104-2.279); p = 0.013] and middle-aged adults (AOR = 1.750; 95% CI (1.075-2.848);p = 0.024] were more likely to adhere to the convenience DP, whereas, the odds of adhering to the convenience pattern was lower among adults residing in rural areas [AOR =0.3161.586; 95% CI (0.219-0.456); p = 0.001].

**Conclusion:**

Socio-economic variables (age, gender, household size and place of residence) were associated with dietary patterns among community dwellers in Nigeria.

**Funding:**

None declared

## Introduction

Nutrition transition has led to changes in dietary patterns, eating habits, and lifestyles.^1^ ^2^ Consumption pattern is gradually undergoing transition from locally produced plant-based staples towards convenience ready-to-eat processed foods,^3^ resulting in an increase in the prevalence of non-communicable diseases in low and middle-income countries. 4 Studies have revealed that nutrition epidemiology has shifted towards the study of dietary patterns (DP) as a way to evaluate the complex relationship between overall diet and disease conditions, rather than laying emphasis on individual nutrients or foods.^1^, ^5^

Sub-Saharan Africa, especially West African countries, is currently experiencing the nutrition transition^4^,^6^,^7^ with dietary changes gradually shifting from a more traditional to westernised diets.^3^, ^4^

In most regions of Nigeria, foods consumed are generally based on root and tuber staples (cassava, yam, maize, plantain) usually served as a group of bolus meals (*eba*, fufu, pounded yam, *amala, semovita* and *tuwo*) 8-10 and consumed with a side dish of vegetable, animal proteins, oils and spices and condiments.^11^

Dietary patterns defined as a group of foods consumed by a given population 12 can be assessed using two broad approaches: *a priori and posteriori*. 13 The a *priori* dietary pattern uses a hypothesis-driven approach based on scoring systems,^13^ whereas the *posteriori* dietary pattern uses an exploratory method which is derived by statistical analysis, such as factor or cluster analysis, and may provide a better description of the actual diet of a specific population group.

Principal component analysis (PCA) is a form of factor analysis which derives linear combinations of foods based on their inter-correlations.^14^ The PCA method is suitable for large population-based studies using food frequency questionnaire (FFQ) data and shows good reproducibility, validity^15^-^17^ and interpretability of the resulting dietary patterns.^15^ Some studies have identified dietary patterns among different population groups; traditional and mixed DPs,^2^ sweet tooth and traditional DPs.^1^ These dietary patterns were named based on the interpretation of the feature of food grouping.^2^

Studies investigating dietary patterns indicate a relationship between socio-demographic variables such as age, gender, marital and socio-economic status, occupation, place of residence and some dietary patterns among adult populations.^18^ However, few researchers have used PCA to establish dietary patterns and associated factors in Nigerian population groups.^19^ A recent investigation among young Nigerian adults using PCA identified four dietary patterns (healthy, bread/drinks, snacks and alcohol DPs),^20^ suggesting that unhealthy dietary patterns were prevalent among the study participants.^20^ The rapid changes in diets and the attendant increase in chronic non-communicable diseases (NCDs), present the need for interventions to promote healthy dietary behaviours among the adult population. Hence, this study identified dietary patterns and evaluated their relationship with socio-demographic factors in community-dwelling adults in Abia State, Nigeria.

## Methods

### Study Design and Participants

This community-based cross-sectional study was carried out among adults (aged 20 to 59 years) in urban (AfaraUkwu and Amuzukwu) and rural (Amawom and Amaoba) areas of Umuahia North and Ikwuano Local Government Areas of Abia State, Nigeria.

### Sample Size and Sampling Technique

The sample size was based on the age distribution of the 2006 census report, where the adult population (> 20 years) was estimated to be 120,153 and 72,635 in Umuahia North and Ikwauno LGAs, respectively. 21 Using the Cochran formula, 22 the sample size was calculated as follows = $\frac{N}{1+N(e{)}^{2}}$, where n = sample size, N= Population size, and e= Level of precision (0.05). An additional 20% was added to make up for possible dropouts in Ikwuano (438) and Umuahia North (439) LGAs. To give an estimated sample size of 877. A three-stage sampling technique was employed to select the communities and adult participants. Umuahia North and Ikwuano LGAs were clustered into urban and rural communities. Two urban and rural communities were randomly selected from each cluster. Simple random sampling was employed to select participants until the required sample size was attained. Pregnant, lactating females and non-Igbo indigenes (male and female adults) residing in the communities were excluded from the study to ensure a homogenous population with similar cultural foods. All eligible participants who agreed to participate were provided oral informed consent before commencing with data collection procedures.

### Data Collection

#### Socio-demographic Factors

A structured questionnaire was used to obtain information on socio-demographics (age, sex, place of residence, and household size) and socio-economic status (educational level, monthly income). Four trained research assistants administered questionnaires to mothers in English and Igbo languages.

#### Dietary Assessment

A validated, culture-sensitive qualitative food frequency questionnaire (FFQ) consisting of over 100 food items commonly consumed in Southeast Nigeria was used to obtain information on food consumption in the last seven days. The foods in the FFQ were adapted from those previously used in a study of the young adult population^20^ and the Nigerian consumption survey.^23^ Participants were asked to recall how often they had consumed a particular food item before the assessment. Consumption frequencies ranging from 0 to 7 were assigned to each food item. Trained research assistants collected all data during a single interview.

#### Dietary Pattern

The over 124 food items obtained from the FFQ were regrouped into 10 sub-groups (Table 1) for use in principal component analysis (PCA) by adding frequencies of food items belonging to similar food groups, i.e. foods with shared nutritional value and culinary preparation.

**Table 1:** Food groups used in factor analysis based on their culinary preparation and nutritional value (The local names in italics are in Igbo language)

	Food groups	Description
**1**	Starchy staples	Yam , Water yam, Sweet potatoes, Irish potatoes, Cocoyam, Garri, Fufu, Abacha, Ripe plantain, Unripe plantain, Pounded yam
**2**	Plant protein	Beans, Bean balls (*akara*), Bean pudding (moi-moi), Green peas, Bambara nut (*okpa*), Black beans (*akidi*), Bread fruit (*ukwa*), Oil bean (*ugba*), Soyabean, Melon (*Egusi*), Groundnut (cooked or roasted), *Fio-fio*
**3**	Animal Protein	Cray fish, Tin fish (sardine, Titus, geisha), dried fish, stock fish, Prawn (*oporo*), Fresh fish (mackerel, *scumbia*), Crab, Beef , Pork, Goat meat, Snail (*ejula*), Periwinkle (isam), Turkey, Chicken, Egg, Eggs, Chicken, turkey
**4**	Dairy based beverages	Tea, Coffee, Cocoa/chocolate, ice cream, yoghurt, milk
**5**	Fruits	Pawpaw, Pineapple, Water melon, Apple, Banana, Avocado pear (*ube bekee*), Plum, Cashew, Coconut, Sour-sop, Guava, Bush mango (*ugiri*), Orange, African star apple (*udara*), Velvet tamarind (*icheku*), Native pear (*ube*), Garden egg (*Anara*), Tangerine, Mango, Grape, Pepper fruit (*mmimi*)
**6**	Alcoholic beverages	Wine, Beer, Whisky/brandy/gin, palm wine
**7**	Processed cereals and grains	Breakfast cereal (cornflakes, goldenmorn), Custard, Pap, *joro*, Wheat, Millet, White corn, Yellow corn, Oats, Pop corn, Rice , Spaghetti, Noodles
**8**	Pastries and soft drinks	Biscuits, cake, meat pie, buns, egg roll, chin chin, puff puff, pancake, Bread, Malt drinks, Orange juice, Mixed fruit juices (canned or packed), Carbonated drinks (cocoa-cola, Pepsi, fanta. Etc)
**9**	Vegetable based soups and sauces	Okro, Spinach, Water leaf, Bitter leaf (onugbo), Scent leaf(*nchuanwu*), Hot leaf (*uziza*), *Ukazi*, Amaranthus (green), Curry leaf, Fruited pumpkin (*ugu*), Uha, Garden egg leaf (*akwukwo Anara*), *Nturukpa*
**10**	Other vegetables	Fresh tomatoes, Pepper, Cabbage, Carrot, Cucumber, Onions, Spring onions, Celery, Green pepper,

#### Data and Statistical Analysis

The socio-demographic and economic variables were classified as follows: Age was divided into two categories: ≤40 (reference group) and >40 years. The place of residence was categorised as rural and urban (reference group). Educational level was classified as having no formal education (reference group) or being educated. Household size was classified as ≤3 (reference group) and >3. Income levels were divided into <#30,000 (reference group), #30,000 to 100,000 and >#100,000.

**Figure 1:**
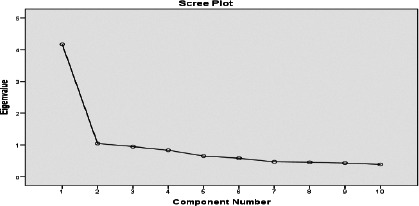
Scree plot for identification of dietary patterns by principal component analysis

Data were analysed using Statistical Package for Social Sciences (SPSS) Version 20 (IBM Inc.). Categorical variables were expressed as frequencies and percentages, while means and standard deviations were used for continuous variables. Bivariate analyses were performed using Chi-square for categorical variables. Dietary patterns were analysed using PCA with orthogonal varimax rotation based on the 10 food groups (Figure 1). The factor analysis required meeting some prerequisites. Kaiser-Meyer-Olkin (KMO) and Bartlett's test of sphericity were used to determine the adequacy of the data set for factorial analysis. The number of dietary patterns/factors to be extracted was based on the eigenvalues greater than 1.0. In addition, the identification of break-point in the scree plot was used to confirm the adequacy of the number of factors retained in the analysis.^25^ Food groups with factor loadings ≥ 0.3 were considered to make significant contributions^1^,^26^ to a particular pattern and retained to aid the interpretation of results.^26^, ^27^ Positive loading indicates that the dietary variable is positively associated with the factor, whereas negative loading reflects an inverse association.

Dietary patterns were labelled based on the composition of food groups, nutritional value, similarity of diets and culinary preparations of the foods in each pattern. The factor scores of the identified patterns were used for further analysis. Pattern-specific factor scores for each participant were calculated as the sum of food factor loading coefficients and the standardised consumption of the foods related to the dietary pattern. The pattern-specific factor scores were divided into four quartiles^1^, ^28^ and further categorised into low adherence (1st, second, and third quartiles) and high adherence (4th quartile), given that the higher the score, the greater the adherence to the pattern.^29^ Bivariate and multivariable logistic regression analyses were used to compare the two selected quartiles (Q1-Q3 and Q4) in the dietary patterns with associated socio-demographic factors. Results were expressed as odds ratios (OR) and 95% confidence intervals (95%CI). Statistical significance was accepted at P< .05.

### Ethical Approval

The Health Research Ethics Committee (HREC) of the Federal Medical Centre, Umuahia, granted ethical approval for the study (reference number: FMC/QEH/G.596/Vol.10/447 and FMC/QEH/G.596/Vol.10/448). All participants read and gave informed consent before the interview.

## Results

### Basic Characteristics of the Participants

Of the total sample (868), males and females accounted for almost equal proportions (49% and 51%, respectively). The majority (70.3%) were less than 40 years old and had a household size greater than three (74.5%). More than half (57.5%) were married, and 50.6% reside in urban areas. More than half (57.4%) earned monthly incomeless than #30,000. Approximately 86.6% are educated, and 64.6% are unskilled workers (Table 2).

**Table 2:** Socio-economic characteristics and Body Mass Index of the adults (n=868)

Characteristics	n (%)
**Sex**	
**Male**	425 (49)
**Female**	443 (51)
**Age (years)**	
**<40 (young adults)**	610 (70.3)
**40 -59 (middle-aged adults)**	258 (29.7)
**Place of residence**	
**Rural**	429 (49.4)
**Urban**	439 (50.6)
**Marital status**	
**Single**	369 (42.5)
**Married**	499 (57.5)
**Education** *	
**Low-level education**	116 (13.4)
**High level educated**	752 (86.6)
**Income**	
**<#30,000**	498 (57.4)
**#30,000 to 100,000**	340 (39.2)
**>#100,000**	30 (3.5)
**Occupation**	
**Employed**	697 (80.1)
**Unemployed**	171 (19.7)
**Household size**	
**≤ 3**	221 (25.5)
**>3**	647 (74.5)
**BMI**	
**Underweight**	30 (3.5)
**Normal**	496 (57.1)
**Overweight/obese**	342 (39.4)

* Low level education=primary school, High level education =secondary and tertiary

### Dietary Patterns of the Adults

The results of the PCA are presented in Table 3. The value of the KMO was 0.887, and Bartlett's test for sphericity gave a value of 2594.115 (p= 0.001). The results of the KMO and the Bartlett tests showed that the data were suitable for factorial analysis.

**Table 3:** Dietary patterns and their component factor loadings

Food groups	Dietary patterns with factor loadings	
	Traditional DP	Convenience DP
**Starch and tubers**	0.610	0.067
**Plant proteins**	0.677	0.288
**Animal proteins**	0.741	0.135
**Dairy-based beverages**	0.666	**-0.200**
**Fruits**	0.778	**-0.252**
**Alcohol**	0.255	0.406
**Processed cereals and grains**	0.586	0.465
**Pastries and soft drinks**	0.605	0.324
**Vegetables-based soups and sauces**	0.684	**-0.433**
**Other vegetables**	0.702	**-0.403**
**% of variance explained** *	41.6	10.4
**KMO**	0.887	
**Bartlett's test**	2594.115	(p= 0.001)

Extraction method, principal component analysis. Rotation method, varimax with Kaiser normalization, KMO: Kaiser-Meyer-Olkin,

* the total explained variation by all factors is 52.0%, food groups with factor loadings below 0.3 contributed little to the dietary patterns, negative factor loading are highlighted in bold

According to the scree plot results (Figure 1), two DPs (traditional and convenience) were identified, and they explained 52% of the variability in the diets of the adults (Figure 1). The first DP was named “traditional” and explained 41.6% of the participants' food intake variability. This DP had high positive factor loadings for most foods; starches and tubers, plant and animal proteins, dairy-based beverages, fruits, cereals, pastries, vegetables and soups, but low factor loading (<0.3) for alcohol. The second DP, called “convenience”, explained the remaining 10.4% variance. This pattern was characterised by high positive loadings for processed cereals and grains, alcohol, pastries and soft drinks and negative loadings for fruits, dairy-based beverages, vegetables, soups, and other vegetables (Table 2). A particular pattern will principally depend on the individual foods that make up the patterns in derived factors.^1^

### Factors Associated with Dietary Patterns

Table 4 shows the factors associated with high adherence to traditional and convenience dietary patterns using bivariate analysis. High adherence to traditional patterns was found among adults with household size greater than 3 (p=0.01). In contrast, high adherence to convenience pattern was common among males (p=0.003), middle-aged adults (p<0.001), rural residents (p=0.003), singles (p<0.001), educated (p=0.003), employed (p=0.043) and household size greater than 3 (p<0.05).

**Table 4:** Bivariate analysis of the factors associated with high adherence to traditional and convenience dietary patterns among adults

Characteristics	Traditional		Convenience	
	High (%)	P value	High (%)	P value
**Gender**				
**Male**	106	0.969	125	0.003*
**Female**	111		92	
**Age (years)**				
**<40**	150	0.668	182	<0.001*
**40** – **59**	67		35	
**Place of residence**				
**Urban**	121	0.078	60	<0.001*
**Rural**	96		157	
**Marital status**				
**Single**	83	0.142	122	<0.001*
**Married**	134		95	
**Education** *				
**Low-level education**	29	1.000	14	<0.001*
**High-level education**	188		203	
**Monthly income**				
**<#30,000**	131	0.637	138	0.100
**#30,000 to 100,000**	80		73	
**>#100,000**	6		6	
**Occupation**				
**Employed**	173	0.805	164	0.043*
**Unemployed**	44		53	
**Household size**				
**≤ 3**	42	0.010*	67	0.034*
**>3**	175		150	

* Factors are statistically significant, according to minimum wage value in 2019 of #30,000, approximated to $83, *Low-level education=primary school, High-level education =secondary and tertiary

Table 5 describes the multivariate regression analysis of socio-demographic factors associated with high adherence to traditional and convenience patterns. Larger households (>3) had lower odds of adhering to the high traditional DP [AOR =0.633; 95% CI (0.429-0.934); p = 0.021] compared with smaller households (≤ 3). Female participants were about twice more likely to adhere to the convenience DP compared to males [AOR =1.586; 95% CI (1.104-2.279); p = 0.013]. Likewise, middle-aged adults (AOR = 1.750; 95% CI(1.075-2.848);p = 0.024] were about twice more likely to adhere to the convenience DP compared to younger adults (≤ 40 years). On the other hand, the odds of adhering to the convenience pattern were lower among adults residing in rural areas [AOR =0.3161.586; 95% CI (0.219-0.456); p = 0.000] compared to urban dwellers [AOR =0.3161.586; 95% CI (0.219-0.456); p = 0.001].

**Table 5:** Multivariate logistic regression exploring socio-demographic factors associated with high adherence to traditional and convenience patterns

Factors	Dietary patterns			
	Traditional		Convenience	
	AOR (95% CI)	P value	AOR (95% CI)	P value
**Sex**				
**Male**	Ref		Ref	
**Female**	1.185 (0.844-1.663)	0.326	1.586 (1.104-2.279)	0.013*
**Age (years)**				
**Young adults (≤40)**	Ref		Ref	
**Middle-aged adults (>40)**	1.085 (0.719-1.637)	0.699	1.750 (1.075-2.848)	0.024*
**Place of residence**				
**Urban**	Ref		Ref	
**Rural**	1.286 (0.917-1.804)	0.146	0.316 (0.219-0.456)	0.001*
**Marital status**				
**Single**	Ref		Ref	
**Married**	0.720 (0.481-1.077)	0.110	1.078 (0.716-1.622)	0.719
**Education** **				
**Low level education**	Ref		Ref	
**High level education**	0.846 (0.513-1.396)	0.513	0.606 (0.319-1.150)	0.319
**Income**				
**<#30,000**	Ref		Ref	
**#30,000 to 100,000**	1.541 (0.601-3.953)	0.368	1.345 (0.509- 3.551)	0.550
**>#100,000**	1.243 (0.484-3.198)	0.651	1.076 (0.405-2.860)	0.883
**Occupation**				
**Employed**	Ref		Ref	
**Unemployed**	0.861 (0.567-1.308)	0.482	1.007 (0.658-1.543)	0.974
**Household size**				
**≤ 3**	Ref		Ref	
**>3**	0.633 (0.429-0.934)	0.021*	1.046 (0.727-1.506)	0.809

* Factors are statistically significant

** Low level education=primary school, High level education =secondary and tertiary

## Discussion

To the best of our knowledge, this is the first study to empirically derive dietary patterns and explore their association with socio-demographic factors among a representative sample of community-dwelling adult population in Nigeria using principal component analysis. Dietary patterns of adults have been characterised using PCA in previous studies in Africa and elsewhere. 18, 24, 30, 31 However, published data for adults in Nigeria is sparse. Two main dietary patterns emerged; “Traditional” and “Convenience”. The identified dietary patterns “traditional” and “convenience” were similar to those reported previously among adults in other studies^18^,^24^ ^30^,^31^,^32^ Some of the identified patterns in previous studies using PCA were named: a healthy DP, mixed DP^20^,^24^ and vegetable DP (high in fruits, vegetables and fish, low in saturated fats and refined sugars).^32^ Another is the western DP, often referred to as unhealthy DP,^33^ varied DP,^34^ risk DP, modern DP,^30^ urban DP, modified DP,^30^ and transitional DP,^31^ which are high in snacks and other unhealthy energy-dense food.^32^

The “traditional” pattern in this study explained the largest variability in the participants' food intake and is highly mixed with processed foods, as observed in other studies among adult populations in Africa.^20^,^30^,^31^,^32^ The traditional pattern showed a distinctive pattern that follows a traditional way of food intake among the *Igbos* in southeast Nigeria, which comprises eating starchy staples (root, tubers and grains) served with soups/sauces, vegetables, animal source foods and other spices and condiments. 11 Thus, the traditional dietary pattern observed in the present study population reflects real-life dietary behaviours consistent with adult populations in the southeastern part of Nigeria.

The traditional patterns have also been identified in various studies.^18^,^30^,^31^,^32^ Nigeria is blessed with traditional and indigenous food crops, which are frequently seasonal and can greatly improve dietary diversity and food security. However, these are often neglected and underutilised.^4^ Among large consumer groups, diets have changed in the past decades in response to preferences for convenience foods.^19^

Among African populations, traditional diets are reportedly healthier than non-traditional ones, even though micronutrient intakes must improve.^35^ The nutrition transition is reported to result in less consumption of local indigenous foods despite the potential health benefits of these foods.^4^ The use of neglected and underutilised foods and crops is well documented in the literature.^36^ ]. Therefore, a concerted effort should be made to explore their use and include them more in diets.

The convenience pattern identified in this study appears to reflect the dietary changes during the nutrition transition process, characterised by Western foods such as high-fat foods, refined sugars, processed cereals, grains, snacks, soft drinks and alcohol.^3^, ^37^ The convenience patterns in this study did not only include a high intake of processed cereals, grains, pastries and soft drinks but also a high intake of alcohol, which is known to contribute empty calories to the body. Consumption of unhealthy diets is at the root of all forms of malnutrition and drives problems such as widespread micronutrient deficiencies and growing rates of diet-related NCDs.^19^ The results serve as a basis for educating and counselling people about healthy diets and promoting the consumption of indigenous foods among adults.

The identified dietary patterns were associated with socio-demographic factors such as household size, age, gender and place of residence. Consumption of less healthy foods has previously been associated with lower socio-economic status.]^38^ In this study, the odds of adhering to the high traditional pattern were lower among larger household participants. A study showed that households with fewer persons had better nutrition outcomes. 39 This can be explained by the fact that as the size of the household increases, the quality and quantity of food consumed may be reduced, thereby increasing the risk of food insecurity and poor nutrient intake. Also, families with larger household sizes may rely on convenience foods to manage family food resources since they are cheaper and readily available. Furthermore, the concern about filling the stomach in the face of dwindling resources rather than the nutritional value of foods may predispose adults from large households to purchase and consume foods from the convenience pattern. 40Interventions to promote healthy eating behaviour among adults should therefore focus on strategies to prepare healthy and quick meals that can still meet the nutritional needs of household members.^41^

With respect to age, middle-aged adults (>40 years) were almost twice more likely to consume foods from the convenience pattern in agreement with previous reports.^42^ It could be that as middle-aged adults engage in various occupations, consumption of fast foods may likely increase. Consistent with our result, the purchase of ready-made meals and processed food products is on the rise among the working-class population in Nigeria.^4^, ^43^ and could portend great danger since it might favour an increase in obesity and other NCDs among the adult population. Again, changes in eating habits, food purchase behaviour and time devoted to food preparation often lead to the widespread consumption of processed and fast foods and foods consumed outside the home by working-class people.^44^ Contrary to our results, some other studies found that older individuals (>60 years) demonstrated high adherence to healthy DP. 45 in contrast to this present study. Therefore, The findings underscore the need to provide strategies to improve healthy dietary behaviours among adults.

Results from this study suggest that high adherence to the convenience pattern is associated with being a female. This may reflect the nutrition transition, which has resulted in changes in the eating habits of both men and women, even from a younger age.^46^ Consistent with our study, female adolescents in South Africa were reported to consume fast foods more often than males. 47 This eating behaviour was attributed to the fact that females stay at home more and are more inactive than males, thus leading to more calorie intake and weight gain among women.^48^]In contrast, a healthier DP was related to the female sex among Bubis adults in Equatorial Guinea.^49^ Furthermore, the female sex was associated with the traditional DP among adults in Burkina Faso.^30^

The present study reinforces that dietary patterns are closely related to the place of residence, with rural dwellers having lower odds for the convenience DP (AOR =0.3161.586; 95% CI (0.219-0.456). This is consistent with that reported in rural areas of Ghana, which showed that the diets of adults were richer in carbohydrates and traditional foods.^20^ Rural dwellers have a greater abundance of starchy staples, fruits and vegetables, and this may increase access and availability of traditional foods than fast foods. In addition, poor purchasing power, bad roads and poor transport system could further reduce rural dwellers' availability and consumption of convenience foods.

A study reported that urban residence is associated with unhealthy dietary patterns.^4^ The emerging trends in unhealthy dietary practices with increased consumption of high-calorie foods, especially among the urban population, could reflect the development level of countries and the process of nutrition transition.^4^ It is also reported that migration of people from rural to urban areas results in significant changes in dietary habits, leading to easy-to-prepare and energy-dense processed foods high in fat, sugar and salt.^4^ This is the case with Nigeria, where there is an increase in fast food outlets serving meals with high salt, fat and sugar content. 4 This study's other independent variables (income and educational level) did not show significant associations as those observed in other studies.^18^

### Limitations

This study is limited by its cross-sectional nature, which does not allow for establishing causal associations. However, the study aimed to investigate associations rather than causality. Again, the use of a qualitative FFQ, did not allow for the calculation of nutrient intake, however, the qualitative FFQ has been shown to indicate the intake pattern in epidemiological studies and helped to minimise recall bias using a 7-day recall period. In addition, the sample was drawn from only one geopolitical region; thus, results may not be generalisable to community dwelling adults in other regions of the country. Comparison of DPs across populations in Sub-Saharan African countries is challenging due to the subjective nature of the applied exploratory methods, from the formation of the food groups to the number of factors that are finally retained.^20^ Despite these limitations, our findings serve as a basis for future studies on dietary patterns and associated factors among community dwelling adults in other geopolitical regions of the country.

## Conclusion

The two dietary patterns (traditional and convenience) identified were associated with the adults' socio-demographic characteristics. Larger household size was less associated with the traditional DP. The convenience DP was associated with female gender and older age, whereas rural residence was less associated with convenience DP. Future studies on dietary patterns of other population groups are urgently needed to provide valuable information that will inform the development of appropriate intervention programs that will help promote and improve healthier food consumption and overall health.
